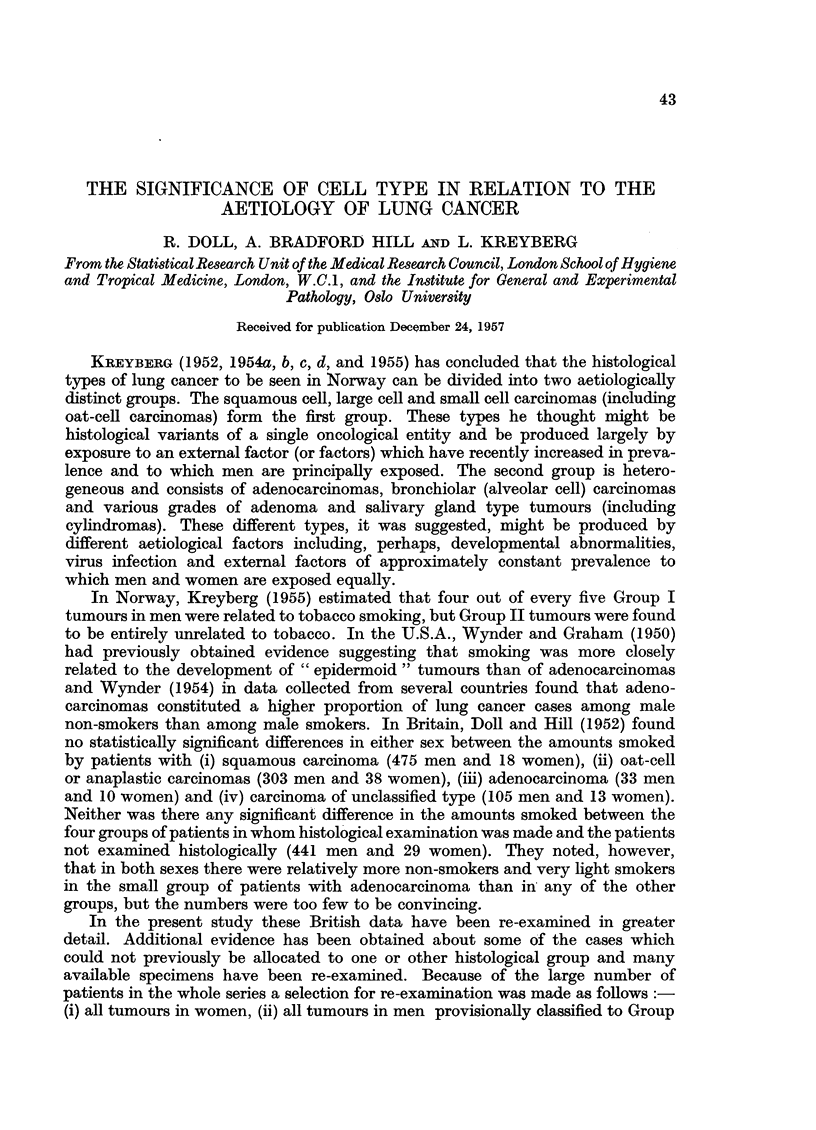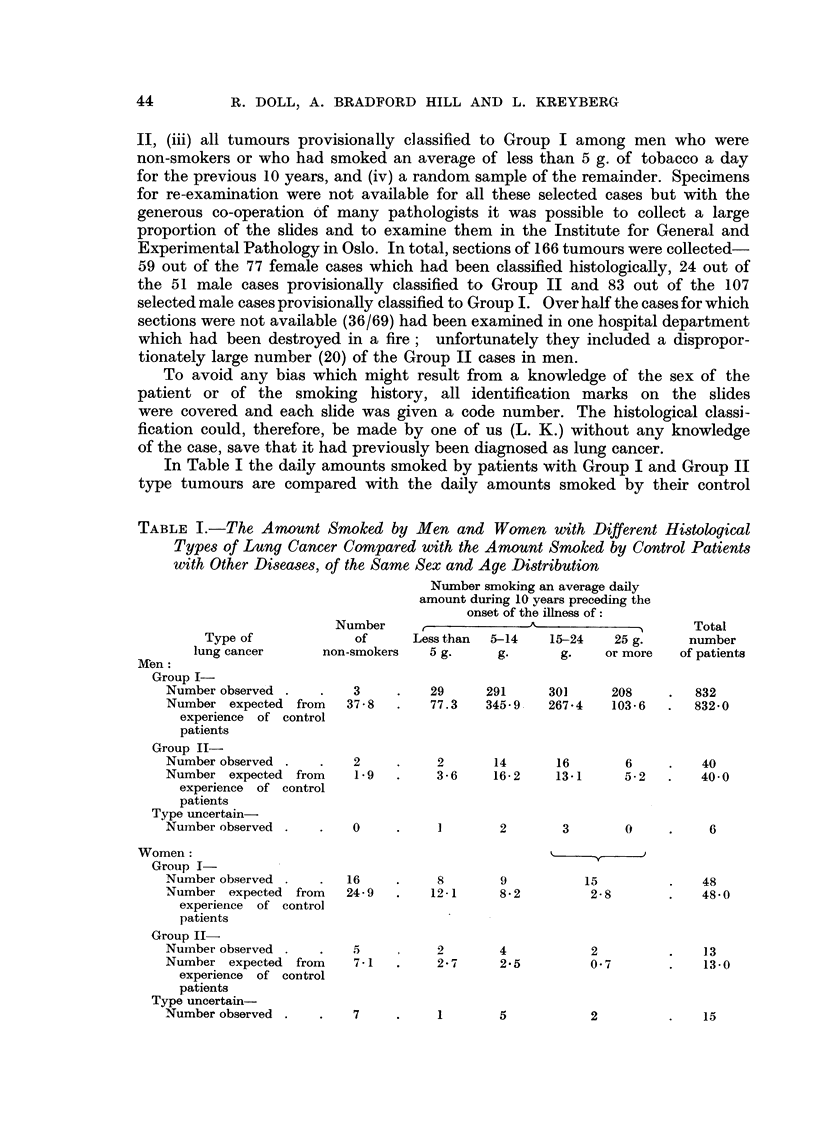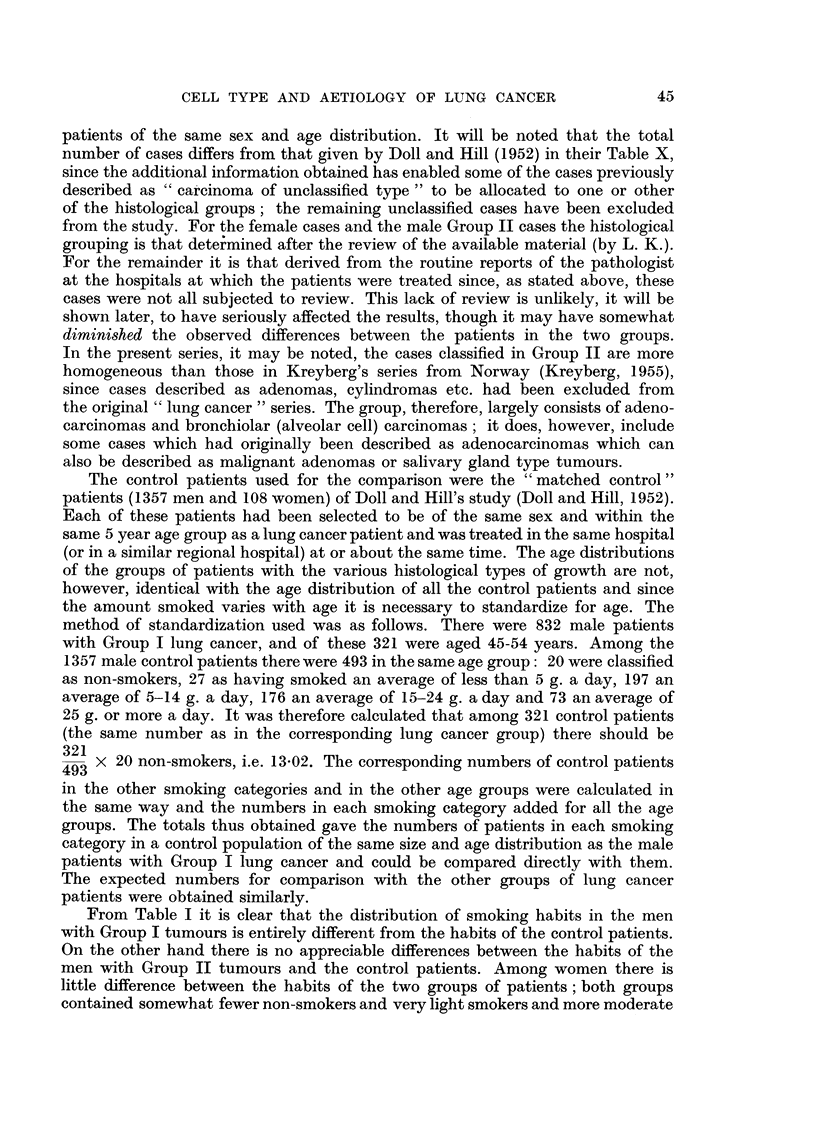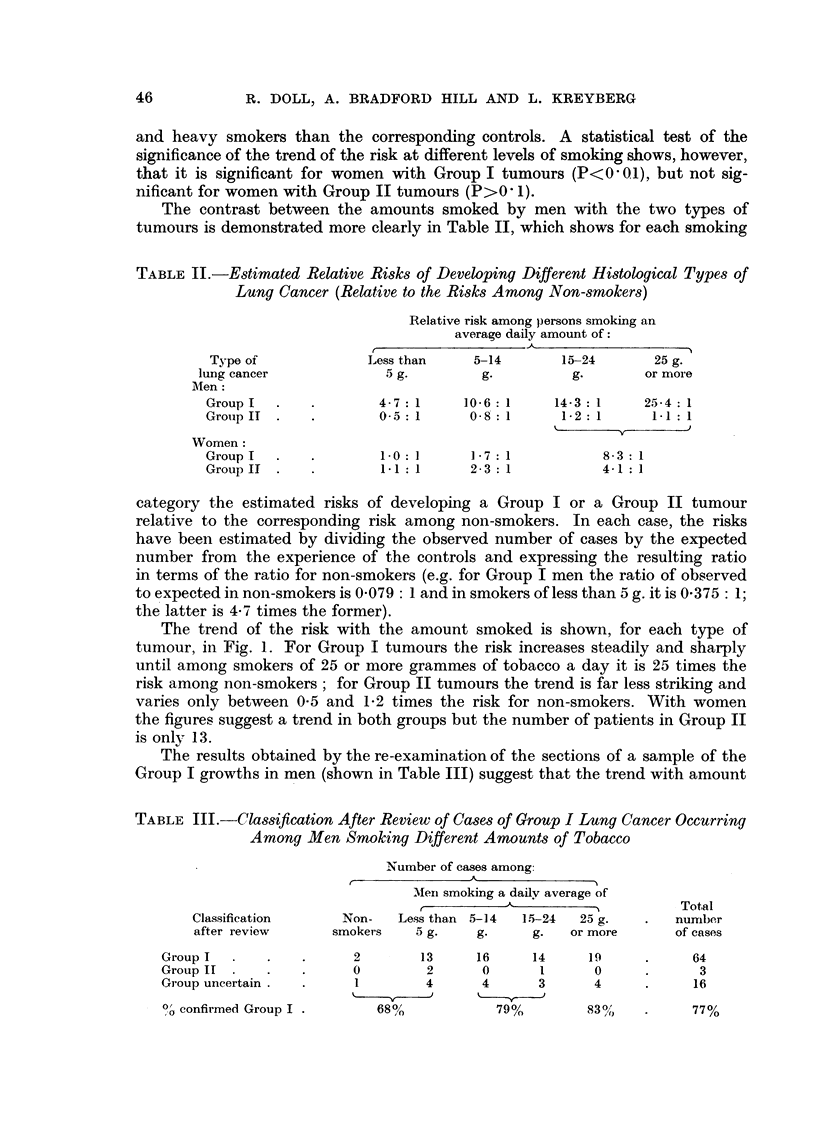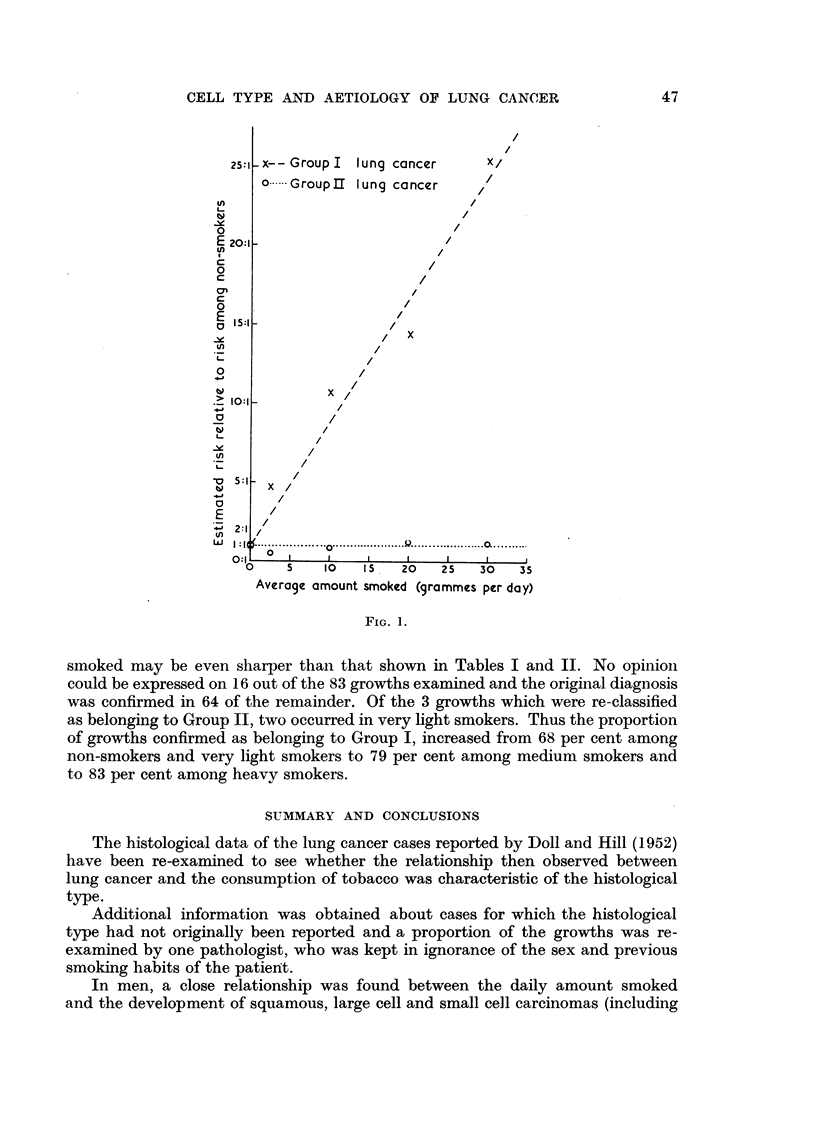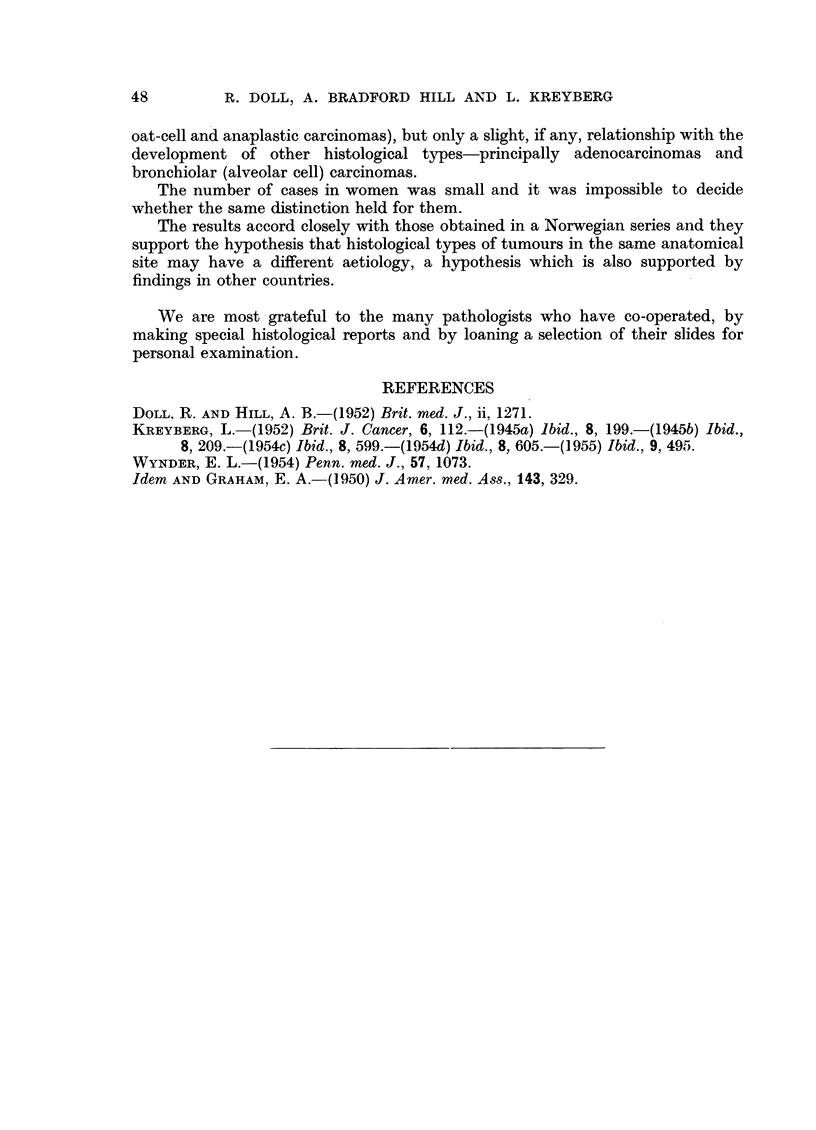# The Significance of Cell Type in Relation to the Aetiology of Lung Cancer

**DOI:** 10.1038/bjc.1957.6

**Published:** 1957-03

**Authors:** R. Doll, A. Bradford Hill, L. Kreyberg


### THE SIGNIFICANCE OF CELL TYPE IN RELATION TO THE AETIOLOGY OF LUNG CANCER

R. DOLL, A. BRADFORD HILL AND L. KREYBERG

*From the Statistical Research Unit of the Medical Research Council, London School of Hygiene and Tropical Medicine, London, W.C.1, and the Institute for General and Experimental Pathology, Oslo University*

Received for publication December 24, 1957

KREYBERG (1952, 1954*a*, *b*, *c*, *d*, and 1955) has concluded that the histological types of lung cancer to be seen in Norway can be divided into two aetiologically distinct groups. The squamous cell, large cell and small cell carcinomas (including oat-cell carcinomas) form the first group. These types he thought might be histological variants of a single oncological entity and be produced largely by exposure to an external factor (or factors) which have recently increased in prevalence and to which men are principally exposed. The second group is heterogeneous and consists of adenocarcinomas, bronchiolar (alveolar cell) carcinomas and various grades of adenoma and salivary gland type tumours (including cylindromas). These different types, it was suggested, might be produced by different aetiological factors including, perhaps, developmental abnormalities, virus infection and external factors of approximately constant prevalence to which men and women are exposed equally.

In Norway, Kreyberg (1955) estimated that four out of every five Group I tumours in men were related to tobacco smoking, but Group II tumours were found to be entirely unrelated to tobacco. In the U.S.A., Wynder and Graham (1950) had previously obtained evidence suggesting that smoking was more closely related to the development of " epidermoid " tumours than of adenocarcinomas and Wynder (1954) in data collected from several countries found that adenocarcinomas constituted a higher proportion of lung cancer cases among male non-smokers than among male smokers. In Britain, Doll and Hill (1952) found no statistically significant differences in either sex between the amounts smoked by patients with (i) squamous carcinoma (475 men and 18 women), (ii) oat-cell or anaplastic carcinomas (303 men and 38 women), (iii) adenocarcinoma (33 men and 10 women) and (iv) carcinoma of unclassified type (105 men and 13 women). Neither was there any significant difference in the amounts smoked between the four groups of patients in whom histological examination was made and the patients not examined histologically (441 men and 29 women). They noted, however, that in both sexes there were relatively more non-smokers and very light smokers in the small group of patients with adenocarcinoma than in any of the other groups, but the numbers were too few to be convincing.

In the present study these British data have been re-examined in greater detail. Additional evidence has been obtained about some of the cases which could not previously be allocated to one or other histological group and many available specimens have been re-examined. Because of the large number of patients in the whole series a selection for re-examination was made as follows :—(i) all tumours in women, (ii) all tumours in men provisionally classified to Group

II, (iii) all tumours provisionally classified to Group I among men who were non-smokers or who had smoked an average of less than 5 g. of tobacco a day for the previous 10 years, and (iv) a random sample of the remainder. Specimens for re-examination were not available for all these selected cases but with the generous co-operation of many pathologists it was possible to collect a large proportion of the slides and to examine them in the Institute for General and Experimental Pathology in Oslo. In total, sections of 166 tumours were collected—59 out of the 77 female cases which had been classified histologically, 24 out of the 51 male cases provisionally classified to Group II and 83 out of the 107 selected male cases provisionally classified to Group I. Over half the cases for which sections were not available (36/69) had been examined in one hospital department which had been destroyed in a fire ; unfortunately they included a disproportionately large number (20) of the Group II cases in men.

To avoid any bias which might result from a knowledge of the sex of the patient or of the smoking history, all identification marks on the slides were covered and each slide was given a code number. The histological classification could, therefore, be made by one of us (L. K.) without any knowledge of the case, save that it had previously been diagnosed as lung cancer.

In Table I the daily amounts smoked by patients with Group I and Group II type tumours are compared with the daily amounts smoked by their control

TABLE I.—*The Amount Smoked by Men and Women with Different Histological Types of Lung Cancer Compared with the Amount Smoked by Control Patients with Other Diseases, of the Same Sex and Age Distribution*

| Type of lung cancer | Number of non-smokers | Number smoking an average daily amount during 10 years preceding the onset of the illness of : Less than 5 g. | 5–14 g. | 15–24 g. | 25 g. or more | Total number of patients |
|---|---|---|---|---|---|---|
| Men : | | | | | | |
| Group I— | | | | | | |
| Number observed | 3 | 29 | 291 | 301 | 208 | 832 |
| Number expected from experience of control patients | 37·8 | 77.3 | 345·9 | 267·4 | 103·6 | 832·0 |
| Group II— | | | | | | |
| Number observed | 2 | 2 | 14 | 16 | 6 | 40 |
| Number expected from experience of control patients | 1·9 | 3·6 | 16·2 | 13·1 | 5·2 | 40·0 |
| Type uncertain— | | | | | | |
| Number observed | 0 | 1 | 2 | 3 | 0 | 6 |
| Women : | | | | 15 g. or more (15–24 g. and 25 g. or more combined) | | |
| Group I— | | | | | | |
| Number observed | 16 | 8 | 9 | 15 | | 48 |
| Number expected from experience of control patients | 24·9 | 12·1 | 8·2 | 2·8 | | 48·0 |
| Group II— | | | | | | |
| Number observed | 5 | 2 | 4 | 2 | | 13 |
| Number expected from experience of control patients | 7·1 | 2·7 | 2·5 | 0·7 | | 13·0 |
| Type uncertain— | | | | | | |
| Number observed | 7 | 1 | 5 | 2 | | 15 |

patients of the same sex and age distribution. It will be noted that the total number of cases differs from that given by Doll and Hill (1952) in their Table X, since the additional information obtained has enabled some of the cases previously described as " carcinoma of unclassified type " to be allocated to one or other of the histological groups ; the remaining unclassified cases have been excluded from the study. For the female cases and the male Group II cases the histological grouping is that determined after the review of the available material (by L. K.). For the remainder it is that derived from the routine reports of the pathologist at the hospitals at which the patients were treated since, as stated above, these cases were not all subjected to review. This lack of review is unlikely, it will be shown later, to have seriously affected the results, though it may have somewhat *diminished* the observed differences between the patients in the two groups. In the present series, it may be noted, the cases classified in Group II are more homogeneous than those in Kreyberg's series from Norway (Kreyberg, 1955), since cases described as adenomas, cylindromas etc. had been excluded from the original " lung cancer " series. The group, therefore, largely consists of adenocarcinomas and bronchiolar (alveolar cell) carcinomas ; it does, however, include some cases which had originally been described as adenocarcinomas which can also be described as malignant adenomas or salivary gland type tumours.

The control patients used for the comparison were the "matched control" patients (1357 men and 108 women) of Doll and Hill's study (Doll and Hill, 1952). Each of these patients had been selected to be of the same sex and within the same 5 year age group as a lung cancer patient and was treated in the same hospital (or in a similar regional hospital) at or about the same time. The age distributions of the groups of patients with the various histological types of growth are not, however, identical with the age distribution of all the control patients and since the amount smoked varies with age it is necessary to standardize for age. The method of standardization used was as follows. There were 832 male patients with Group I lung cancer, and of these 321 were aged 45-54 years. Among the 1357 male control patients there were 493 in the same age group : 20 were classified as non-smokers, 27 as having smoked an average of less than 5 g. a day, 197 an average of 5–14 g. a day, 176 an average of 15–24 g. a day and 73 an average of 25 g. or more a day. It was therefore calculated that among 321 control patients (the same number as in the corresponding lung cancer group) there should be $\frac{321}{493} \times 20$ non-smokers, i.e. 13·02. The corresponding numbers of control patients in the other smoking categories and in the other age groups were calculated in the same way and the numbers in each smoking category added for all the age groups. The totals thus obtained gave the numbers of patients in each smoking category in a control population of the same size and age distribution as the male patients with Group I lung cancer and could be compared directly with them. The expected numbers for comparison with the other groups of lung cancer patients were obtained similarly.

From Table I it is clear that the distribution of smoking habits in the men with Group I tumours is entirely different from the habits of the control patients. On the other hand there is no appreciable differences between the habits of the men with Group II tumours and the control patients. Among women there is little difference between the habits of the two groups of patients ; both groups contained somewhat fewer non-smokers and very light smokers and more moderate and heavy smokers than the corresponding controls. A statistical test of the significance of the trend of the risk at different levels of smoking shows, however, that it is significant for women with Group I tumours ($P<0\cdot01$), but not significant for women with Group II tumours ($P>0\cdot1$).

The contrast between the amounts smoked by men with the two types of tumours is demonstrated more clearly in Table II, which shows for each smoking

TABLE II.—*Estimated Relative Risks of Developing Different Histological Types of Lung Cancer (Relative to the Risks Among Non-smokers)*

| Type of lung cancer | Relative risk among persons smoking an average daily amount of: Less than 5 g. | 5–14 g. | 15–24 g. | 25 g. or more |
|---|---|---|---|---|
| Men: | | | | |
| Group I | 4·7 : 1 | 10·6 : 1 | 14·3 : 1 | 25·4 : 1 |
| Group II | 0·5 : 1 | 0·8 : 1 | 1·2 : 1 | 1·1 : 1 |
| Women: | | | | |
| Group I | 1·0 : 1 | 1·7 : 1 | 8·3 : 1 (15 g. or more) | |
| Group II | 1·1 : 1 | 2·3 : 1 | 4·1 : 1 (15 g. or more) | |

category the estimated risks of developing a Group I or a Group II tumour relative to the corresponding risk among non-smokers. In each case, the risks have been estimated by dividing the observed number of cases by the expected number from the experience of the controls and expressing the resulting ratio in terms of the ratio for non-smokers (e.g. for Group I men the ratio of observed to expected in non-smokers is 0·079 : 1 and in smokers of less than 5 g. it is 0·375 : 1; the latter is 4·7 times the former).

The trend of the risk with the amount smoked is shown, for each type of tumour, in Fig. 1. For Group I tumours the risk increases steadily and sharply until among smokers of 25 or more grammes of tobacco a day it is 25 times the risk among non-smokers ; for Group II tumours the trend is far less striking and varies only between 0·5 and 1·2 times the risk for non-smokers. With women the figures suggest a trend in both groups but the number of patients in Group II is only 13.

The results obtained by the re-examination of the sections of a sample of the Group I growths in men (shown in Table III) suggest that the trend with amount

TABLE III.—*Classification After Review of Cases of Group I Lung Cancer Occurring Among Men Smoking Different Amounts of Tobacco*

| Classification after review | Number of cases among: Non-smokers | Men smoking a daily average of: Less than 5 g. | 5–14 g. | 15–24 g. | 25 g. or more | Total number of cases |
|---|---|---|---|---|---|---|
| Group I | 2 | 13 | 16 | 14 | 19 | 64 |
| Group II | 0 | 2 | 0 | 1 | 0 | 3 |
| Group uncertain | 1 | 4 | 4 | 3 | 4 | 16 |
| % confirmed Group I | 68% (non-smokers and less than 5 g.) | | 79% (5–24 g.) | | 83% | 77% |

FIG. 1.

smoked may be even sharper than that shown in Tables I and II. No opinion could be expressed on 16 out of the 83 growths examined and the original diagnosis was confirmed in 64 of the remainder. Of the 3 growths which were re-classified as belonging to Group II, two occurred in very light smokers. Thus the proportion of growths confirmed as belonging to Group I, increased from 68 per cent among non-smokers and very light smokers to 79 per cent among medium smokers and to 83 per cent among heavy smokers.

### SUMMARY AND CONCLUSIONS

The histological data of the lung cancer cases reported by Doll and Hill (1952) have been re-examined to see whether the relationship then observed between lung cancer and the consumption of tobacco was characteristic of the histological type.

Additional information was obtained about cases for which the histological type had not originally been reported and a proportion of the growths was re-examined by one pathologist, who was kept in ignorance of the sex and previous smoking habits of the patient.

In men, a close relationship was found between the daily amount smoked and the development of squamous, large cell and small cell carcinomas (including oat-cell and anaplastic carcinomas), but only a slight, if any, relationship with the development of other histological types—principally adenocarcinomas and bronchiolar (alveolar cell) carcinomas.

The number of cases in women was small and it was impossible to decide whether the same distinction held for them.

The results accord closely with those obtained in a Norwegian series and they support the hypothesis that histological types of tumours in the same anatomical site may have a different aetiology, a hypothesis which is also supported by findings in other countries.

We are most grateful to the many pathologists who have co-operated, by making special histological reports and by loaning a selection of their slides for personal examination.

### REFERENCES

Doll, R. and Hill, A. B.—(1952) *Brit. med. J.*, ii, 1271.
Kreyberg, L.—(1952) *Brit. J. Cancer*, **6**, 112.—(1945*a*) *Ibid.*, **8**, 199.—(1945*b*) *Ibid.*, **8**, 209.—(1954*c*) *Ibid.*, **8**, 599.—(1954*d*) *Ibid.*, **8**, 605.—(1955) *Ibid.*, **9**, 495.
Wynder, E. L.—(1954) *Penn. med. J.*, **57**, 1073.
*Idem* and Graham, E. A.—(1950) *J. Amer. med. Ass.*, **143**, 329.